# Identification and evolutionary analysis of insect fibrillin with their closely related family proteins

**DOI:** 10.7717/peerj.21577

**Published:** 2026-08-24

**Authors:** Jiani Chen, Feng Zhou, Xinyu Sun, Zhuanxia Li, Shuning Sun, Yuying Zhang, Lixia Wan

**Affiliations:** College of Life Science, Northwest Normal University, Lanzhou, China

**Keywords:** Fibrillin, Insects, Evolutionary analysis, Protein family

## Abstract

Fibrillins constitute a class of extracellular matrix proteins, and the C-terminal peptide of vertebrate fibrillin members has been identified as a hormone that regulates glucose metabolism. Their evolutionary history has attracted increasing attention recently as the hormonal roles seemingly originated in invertebrates. However, the evolution of fibrillin homologs in invertebrates such as insects, remains poorly understood. In this study, 373 annotated fibrillin sequences (including fibrillin-1, fibrillin-2, and fibrillin-3) from 181 species, 126 genera, 65 families, and 12 insect orders were retrieved and analyzed. Multidimensional analysis of phylogenesis, sequence features, conserved motifs, functional domains, and three-dimensional structures was performed on these sequences to elucidate their evolutionary relationships. Phylogenetic analysis categorized all annotated insects fibrillin sequences into three distinct clades. Among them, only the fibrillin-2 annotated members exhibit clear homology to the vertebrate fibrillin family, having diverged early from a putative ancestral protein together with the other two fibrillin annotated groups. Analysis of cysteine ratio, motif, domain composition, and furin cleavage site also revealed that only insect fibrillin-2 annotated homologs share highly similar sequence and structural features with vertebrate fibrillins. By contrast, the other two insect fibrillin annotated members are more divergent from the canonical fibrillin. Combined analyses of sequence conservation, phylogeny, domain analysis, and three-dimensional structural comparisons demonstrates that insect annotated fibrillin-1 proteins are more closely related to vertebrate fibulin than to canonical fibrillins, whereas insect annotated fibrillin-3 proteins may represent an uncharacterized insect-specific protein family associated with cell adhesion. These two groups are closely related to proteins of fibrillin family and have been misannotated in public database.

## Introduction

The extracellular matrix (ECM) constitutes a three-dimensional network of macromolecules that provides structural support to cells and tissues (Theocharis et al., 2016), and is primarily composed of collagen, fibronectin, laminin, fibrillin and multipleother glycoproteins (Davis & Summers, 2012). Among these components, fibrillin family members represent the primary proteins responsible for the structure and function of 10 nm extensible microfibrils in extracellular matrix and play a pivotal role in its structural organization and functional properties (Sakai, Keene & Engvall, 1986; Keene et al., 1991; Alonso et al., 2022). As collagenase-resistant glycoproteins of approximately 350 kDa, fibrillins are present in both elastin-rich and non-elastin connective tissues (Sakai, Keene & Engvall, 1986). They execute architectural functions in most connective tissues, maintain the structural integrity of individual organ systems, and interact with other ECM proteins to sustain the elasticity of connective tissues (Sakai, Keene & Engvall, 1986; Kielty et al., 2002b; Wagenseil & Mecham, 2009; Summers et al., 2023).

Extant vertebrate fibrillin-1, fibrillin-2, and fibrillin-3 originally evolved from ancestral fibrillins (Piha-Gossack, Sossin & Reinhardt, 2012). Molecular cloning and sequencing of fibrillins have revealed that fibrillin-1, fibrillin-2, and fibrillin-3 share highly homologous primary structures, which consist of eight-cysteine epidermal growth factor-like domains and other domains (Handford et al., 2000). Despite their close sequence homology, these three fibrillin isoforms exhibit distinct expression patterns in developing and adult tissues and perform diverse biological functions (Kielty et al., 2005). Fibrillin-1, the first member of this family isolated from human fibroblast cultures, is mainly distributed in tissues rich in elastic fibers and ubiquitously expressed in mesenchymal cell types (Sakai, Keene & Engvall, 1986), providing support for connective tissue and participating in elastic fiber formation and jointly maintain the structural integrity of the extracellular matrix (Ramirez & Pereira, 1999; Genot et al., 2025). In contrast to fibrillin-1, fibrillin-2 plays a significant role in the early stage of embryonic development, especially in the development of tissues such as the heart and large blood vessels. The expression of fibrillin-2 is largely limited to fetal development, as development matures, its expression level gradually decreases. And mainly regulates the early process of elastic fiber assembly (Zhang, Hu & Ramirez, 1995; Charbonneau et al., 2003). Fibrillin-3 is expressed early in human development (in embryonic and fetal tissues), although it is not expressed in rodents (such as rats and mice) (Sabatier et al., 2011). Notably, it is the only fibrillin found in the perichondral chondrogenic layer, suggesting it may play a unique role in bone growth and development (Corson et al., 2004; Sabatier et al., 2011; Davis & Summers, 2012).

Fibrillins are synthesized as precursor proteins that can be cleaved by furin/PACE-like proteases (Ramirez & Pereira, 1999). In vertebrates, the C-terminal product of furin-cleaved fibrillin-1 and fibrillin-2 exert complex biological functions (Romere et al., 2016), and serve as crucial regulatory factors in maintaining energy metabolism homeostasis. They act on multiple organs *via* diverse downstream signaling pathways and are closely implicated in a wide of metabolic diseases. Additionally, these products are tightly associated with multiple physiological processes, including appetite regulation (Duerrschmid et al., 2017), glucose homeostasis (Li et al., 2019; Yu et al., 2020), and insulin resistance (Romere et al., 2016; Mishra et al., 2021). Asprosin, is a fasting-induced glucogenic hormone, secreted by white adipose tissue in response to starvation (Moradi et al., 2021; Nedeva et al., 2023; Keskin et al., 2025). And a glucogenic hormone discovered through investigations of neonatal progeroid syndrome (NPS), is a C-terminal cleavage product of fibrillin-1, functions similarly to glucagon (Romere et al., 2016; Ovali & Bozgeyik, 2022; Liu et al., 2026). Asprosin is mainly released from adipose tissue into the circulatory system and interacts with the G protein-coupled receptor OLFR734 on the liver surface (Li et al., 2019; Mishra et al., 2022). By activating the cAMP signaling pathway, it rapidly induces glucose production and hepatic glycogen release under fasting and obese conditions, achieving the effect of raising blood sugar and maintaining glucose homeostasis balance (Li et al., 2019; Diao et al., 2023; Liu et al., 2026). In addition, it can cross the blood–brain barrier and act on the hypothalamus, and bind to the surface receptors of hypothalamic neurons, affecting the activity of POMC neurons and NPY/AgRP neurons. Among them, the activation of POMC neurons usually inhibits appetite, while the activation of NYP/AgRP neurons promotes appetite (Hoffmann, Xie & Chopra, 2020; Mazur-Bialy, 2021). In addition, asprosin has also been proven to act on islet *β* cells and skeletal muscle tissue, regulating insulin secretion and insulin sensitivity, and ultimately achieving the effect of raising blood glucose (Romere et al., 2016; Duerrschmid et al., 2017). The C-terminal hormonal peptide of human fibrillin-2, annotated placensin, participates in regulation of metabolism and placental function (Yu et al., 2020; Hu et al., 2023). In zebrafish, this peptide has been demonstrated to stimulate hepatic glucose secretion, promote appetite *via* secretion by gonadal germ cells, and facilitate oocyte maturation and vitellogenin expression (Hu et al., 2023).

So far, homologous proteins of fibrillin have been found in many vertebrates, exhibiting significant conservation in family members and sequences. Studies on fibrillin in vertebrates have shown that three fibrillin family members evolved from an ancestral fibrillin present in invertebrates (Davis & Summers, 2012; Piha-Gossack, Sossin & Reinhardt, 2012). The more definite view is that member 1 evolved first, followed by the formation of members 2 and 3. This pattern has also been supported in many vertebrates, as indeed the differences of member 1 are greater in many species. Although vertebrates are closely related to invertebrates in terms of origin, predecessors have proposed that the ancient ancestor of fibrillin originated from the fibrillin ancestor of invertebrates. However, the evolutionary mechanism, structure and function of this ancestral protein in invertebrates remain unclear. Investigating fibrillin in invertebrates can reveal its primitive form and function during the early stage of biological evolution, thereby providing insights into the evolutionary patterns of fibrillin in these organisms. Insects, a diverse and representative invertebrate group that plays a crucial role in animal evolution, were selected as representatives of invertebrates for study fibrillin.

Invertebrates are generally believed to possess a single ancestral fibrillin gene. However, three different isoforms are currently annotated as “fibrillin” in the NCBI database for many insect species, suggesting possible errors in sequence-based annotation or functional interpretation. Therefore, fibrillins in insects, a representative invertebrate group, were studied and analyzed in this study. The physicochemical properties, phylogenetic, conservative motifs, domains, sequence characteristics, and C-terminal domains of insect fibrillins and related proteins were analyzed to identify family members and clarify their relationships. Our results indicate that insects possess only one genuine fibrillin, corresponding to annotated fibrillin-2. The proteins annotated as fibrillin-1 are more closely related to fibulins in vertebrates, whereas those annotated as fibrillin-3 may represent a previously uncharacterized protein family. These findings clarify the annotation confusion surrounding insect fibrillin-related proteins and provide a basis for further functional studies.

## Materials and Methods

### Sequence collection

Using published vertebrate fibrillin sequences as queires, and insect species retrieved from the InsectBase 2.0 (http://v2.insect-genome.com/) as the target organisms, BLASTP searches was performed against the NCBI non-redundant (NR) database to screen fibrillin protein sequences from target species. Sequences with an *E* value ≤ 1e-10 and annotated with keywords including “fibrillin”, “FBN”, “fibrillin-like” and “similar to fibrillin” were regarded as putative fibrillin sequences. Ultimately, more than 400 fibrillin protein sequences covering 12 insect orders were acquired. All candidate sequences were subjected to multiple sequence alignment using Clustal X, followed by the removal of redundant sequences and biologically implausible sequences originating from genome misassembly, incorrect gene prediction, or pseudogene annotation.

### Multiple sequence alignment and phylogenetic analyses

Multiple sequence alignment of the protein sequences was performed using Clustal Omega available on the EMBL-EBI web server (ebi.ac.uk/jdispatcher/msa/clustalo). To explore the evolution characteristics of insect fibrillins, more than 400 fibrillin annotated proteins covering 12 insect orders were retrieved from diverse insect species. Preliminary phylogenetic analyses (Misof et al., 2014) were conducted to screen the dataset, with severely truncated and highly divergent sequences excluded to minimize potential long-branch attraction. The refined dataset contained 385 sequences derived from 181 species, 126 genera, and 65 families across 12 insect orders and was subjected to maximum likelihood phylogenetic analysis. Ultimately, a phylogenetic tree was constructed based on 373 high-quality sequences using IQ-TREE (http://www.iqtree.org/), with 1,000 Bootstrap replicates for branch support evaluation (Trifinopoulos et al., 2016). The resulting of phylogenetic tree was visualized using the online iTOL v6 (https://itol.embl.de/), and figure presentation was further optimized with Adobe Illustrator 2024.

### Protein sequence analysis

The online tool ExPasy-ProtParam was employed to predict the basic physicochemical properties of protein sequences and conduct statistical analysis of cysteine residue content (Gasteiger et al., 2003). The furin cleavage site Arg-Xaa-Lys/Arg-Arg (R-X-K/R-R) is evolutionarily conserved. Therefore, the presence of this site was regarded as supportive evidence for the identification of bona fide fibrillin sequences (Thomas, 2002). The online tool ProP 1.0 (https://services.healthtech.dtu.dk/services/ProP-1.0/) was used to predict the furin cleavage sites in insect fibrillin protein sequences (Duckert, Brunak & Blom, 2004).

### Conserved motifs and domain analysis

The online MEME Suite 5.5.5 (https://meme-suite.org/meme/tools/meme) was used to identify and analyze conserved motifs. The maximum number of predicted motifs was set to 15, with the optimal motif width constrained to 6–100 amino acid residues. Protein domain was annotated using the Interpro online server (https://www.ebi.ac.uk/interpro/), and domain architectures were visualized with distinct colors and shapes using GPS-IBS 2.0 software (Xie et al., 2022).

### Protein three-dimensional structure prediction

Protein sequences were submitted to the online AlphaFold2 platform (https://colab.research.google.com/github/sokrypton/ColabFold/blob/main/AlphaFold2.ipynb) for three-dimensional structure prediction. The model with the highest predicted local distance difference test (pLDDT) confidence value was selected for subsequent analysis (Varadi et al., 2022). Structural visualization and superposition of the predicted models were performed using PyMOL 4.6.0.

## Results

### Sequence collection of fibrillin annotated proteins in insects

Analysis of the collected insect sequences annotated as fibrillin revealed that these proteins could be classified into three distinct subfamilies. Subsequent functional and evolutionary analyses confirmed that only one subfamily exhibits genuine homology to vertebrate fibrillins, while the other two subfamilies are likely misannotated. Blattodea, Orthoptera, Hymenoptera, and Thysanoptera each possess three annotated fibrillin proteins (fibrillin-1, fibrillin-2, and fibrillin-3), while Hemiptera and Neuroptera harbor only fibrillin-2. Multiple insect orders contain two annotated fibrillin members, including Ephemeroptera (fibrillin-1, fibrillin-2), Odonata (fibrillin-2 fibrillin-3), Lepidoptera (fibrillin-1, fibrillin-2), Coleoptera (fibrillin-2, fibrillin-3), Diptera (fibrillin-1, fibrillin-3), and Phasmatodea (fibrillin-2, fibrillin-3). Notably, Diptera is the only order among the 12 investigated insect orders that lacks the fibrillin-2 homolog (Table S1).

### Insect fibrillin-2 has the typical cysteine composition ratio of fibrillin family proteins

Fibrillins are highly conserved across evolution, and a defining characteristic of this protein family is a cysteine residue content of 12–13% (Hubmacher et al., 2011). Accordingly, the amino acid sequences of representative fibrillin proteins were subjected to physicochemical property prediction using ExPasy-ProtParam (Table 1). Although cysteine distribution is generally conserved among annotated insect fibrillin sequences, fibrillin-2 exhibits a denser cysteine than annotated fibrillin-1 and fibrillin-3 proteins. Specifically, only insect fibrillin-2 possesses a cysteine content of 12–13%, which matches the canonical amino acid profile of the fibrillin family. In comparison, fibrillin-1 contains 8–11% cysteine residues, and fibrillin-3 contains 10–12%, both of which deviate substantially from the typical fibrillin family signature. Furthermore, the theoretical isoelectric points of insect fibrillins range from 4.7 to 5.3, indicating that all are acidic proteins. In terms of sequence characteristics, fibrillin-1 annotated proteins have amino acid length of 917–1,624 aa and corresponding molecular weight of 102.1–180.26 kD; fibrillin-2 annotated proteins range from 2,494–3,022 aa in length, with a molecular weight of 272.86–322.44 kD; fibrillin-3 annotated proteins span 1,425–2,298 aa, with molecular weight of 159.29–251.99 kD. Collectively, these findings reveal distinct physicochemical properties among the three insect fibrillin annotated subtypes.

**Table 1:** Physicochemical property fibrillins in insects.

Protein ID	Name	Order	Species	Protein length (aa)	Molecular weight (kD)	pI	Cysteine content (%)
XP_034240918.1	fibrillin-1	Thysanoptera	*Thrips palmi*	1,437	155.54	5.13	10.1
XP_006558164.2	fibrillin-1	Hymenoptera	*Apis mellifera*	1,624	180.26	5.19	9.7
XP_037876845.1	fibrillin-1	Lepidoptera	*Bombyx mori*	1,499	166.03	5.12	7.8
XP_049844465.1	fibrillin-1	Orthoptera	*Schistocerca gregaria*	1,283	141.45	4.76	10.8
XP_023706876.1	fibrillin-1	Blattodea	*Cryptotermes secundus*	1,473	162.54	4.94	10.9
NP_001284712.1	fibrillin-1	Diptera	*Drosophila melanogaster*	917	102.10	5.34	9.8
XP_034242198.1	fibrillin-2	Thysanoptera	*Thrips palmi*	2,948	315.25	4.97	12.4
CAD7455269.1	fibrillin-2	Phasmatodea	*Timema tahoe*	2,494	272.86	4.67	12.1
XP_003250904.3	fibrillin-2	Hymenoptera	*Apis mellifera*	2,876	315.30	4.84	12.9
XP_037874370.1	fibrillin-2	Lepidoptera	*Bombyx mori*	3,022	322.44	4.75	12.2
XP_008201364.1	fibrillin-2	Coleoptera	*Tribolium castaneum*	2,870	314.10	5.01	12.9
XP_049837718.1	fibrillin-2	Orthoptera	*Schistocerca gregaria*	2,888	314.48	4.87	12.8
XP_023706358.1	fibrillin-2	Blattodea	*Cryptotermes secundus*	2,908	316.73	4.95	12.7
XP_034254429.1	fibrillin-3	Thysanoptera	*Thrips palmi*	2,020	214.58	4.76	10.8
CAD7456482.1	fibrillin-3	Phasmatodea	*Timema tahoe*	2,298	251.99	4.85	10.5
XP_006561358.2	fibrillin-3	Hymenoptera	*Apis mellifera*	2,749	301.53	4.73	11.7
XP_021695535.1	fibrillin-3	Diptera	*Aedes aegypti*	1,425	159.29	5.3	10.7
XP_008199507.2	fibrillin-3	Coleoptera	*Tribolium castaneum*	1,867	207.92	4.76	10.9
XP_023722246.1	fibrillin-3	Blattodea	*Cryptotermes secundus*	2,065	226.53	4.99	11.2

### Insect fibrillins show a unique evolutionary pattern

**Figure 1:**
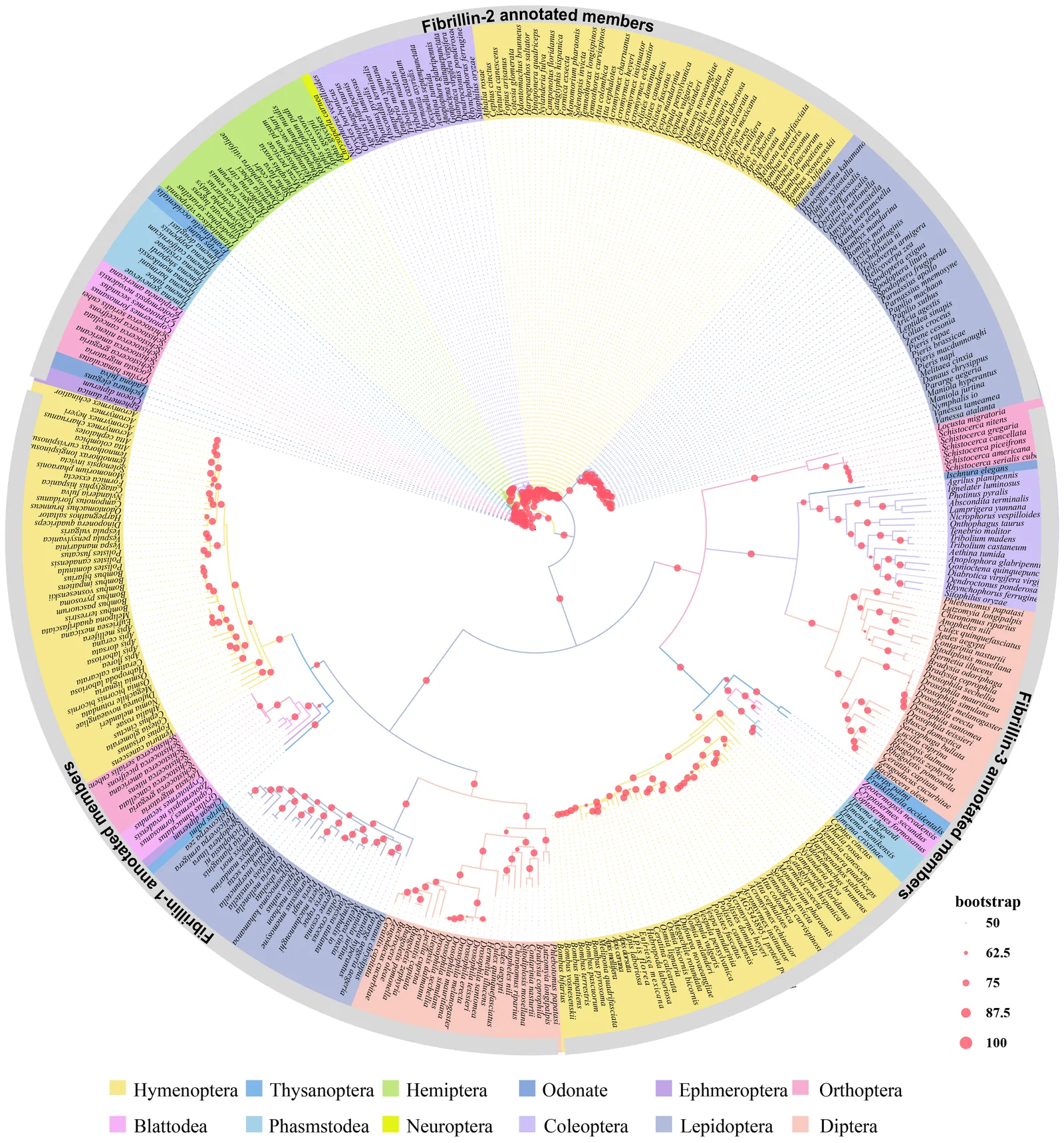
Phylogenetic analysis of insect fibrillin annotated proteins. Maximum likelihood phylogenetic analysis was performed on the final set of 373 sequences from 12 orders 65 families, 126 genera, 181 species. Analyses were conducted using the JTTDCMut+I+G4. Bootstrap values are shown for dots of different sizes. Ranch lengths are proportional to sequence divergence. Different colored areas were used to distinguish insect species from each order.

To facilitate the comparative analysis of insect fibrillins, more than 300 fibrillin sequences were identified covering 12 insect orders (Table 2). The maximum likelihood phylogenetic tree constructed based on 373 sequences indicated that fibrillin-2 may represent the ancestral gene subtype (Fig. 1). Located at the basal clade of the tree, fibrillin-2 emerged early during insect evolution and exhibits an earlier origin than fibrillin-1 and fibrillin-3. The widespread occurrence of fibrillin-2 across multiple insect orders further supports its early emergence and retention during insect radiation, confirming that fibrillin-2 constitutes the bona fide insect fibrillin lineage. The corresponding evolutionary branches show nested phylogenetic relationships: Neuroptera and Coleoptera form a single sub-branch; Thysanoptera and Hemiptera, together with the earlier-diverging Ephemeroptera, Odonata, Orthoptera, and Phasmatodea, constitute two additional sub-branches; while the later-diverging Lepidoptera are located at the terminal branch. Furthermore, the phylogenetic placement of the Lepidoptera and Diptera within the fibrillin-1 and fibrillin-3 clades indicates the recent emergence of these two subtypes in these two insect orders. Additionally, Orthoptera, Blattodea, and Phasmatodea cluster within a single monophyletic branch, demonstrating their close evolutionary homology.

**Table 2:** Summary of fibrillins content in insect species.

Order	Number of family	Number of genus	Number of species	Number of fibrillins
Ephemeroptera	2	2	2	3
Neuroptera	1	1	1	1
Thysanoptera	1	2	2	5
Phasmatodea	1	1	9	13
Odonata	2	2	2	3
Hymenoptera	8	28	46	134
Diptera	12	19	27	51
Lepidoptera	12	27	39	70
Coleoptera	11	19	20	37
Hemiptera	9	18	21	21
Orthoptera	2	3	8	25
Blattodea	4	4	4	10
Total	65	126	181	373

### Motif analysis revealed considerable variations in the number and arrangement of conserved motifs in insect annotated fibrillins

**Figure 2:**
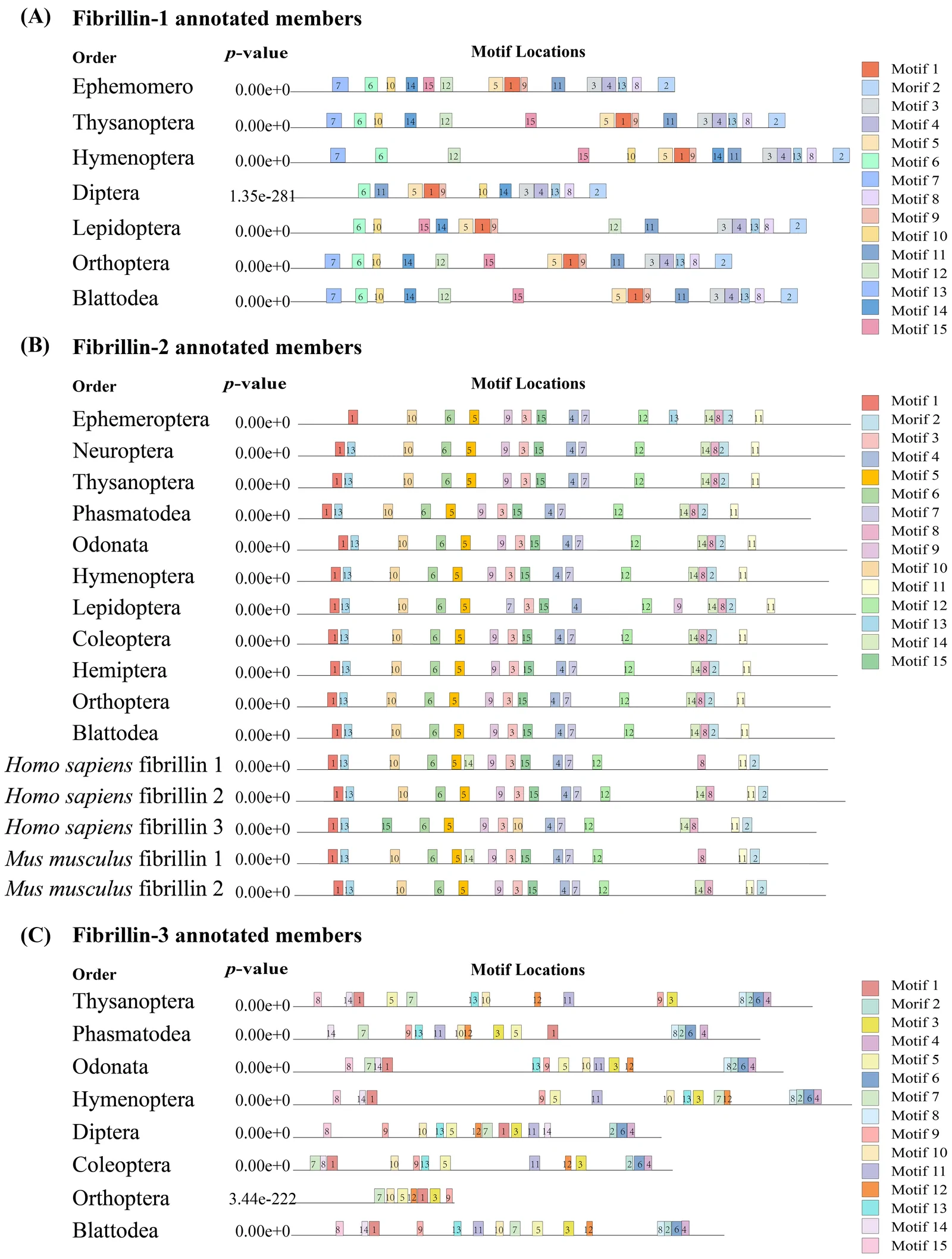
Motif analysis of insect fibrillin annotated members. (A) Conserved motif of the insect fibrillin-1 annotated members, including Ephemeroptera, Thysanoptera, Hymenoptera, Diptera, Lepidoptera, Orthoptrea and Blattodea. (B) Conserved motif of the insect fibrillin-2 annotated members and fibrillins in vertebrates (*Homo sapiens* and *Mus musculus*). (C) Conserved motif of the insect fibrillin-3 annotated members, including 8 orders. The amino acid motifs in annotated fibrillins are displayed in fifteen colored boxes, and black lines indicate amino acid length.

To identify whether insect fibrillin-1 and fibrillin-3 annotated proteins differ from fibrillin-2 annotated members in sequence composition, conserved motif of fibrillin annotated sequences from 12 insect orders were analyzed using the online MEME tool. The results revealed distinct differences in motif composition among annotated fibrillin-1, fibrillin-2, and fibrillin-3 members (Fig. 2). Annotated fibrillin-2 proteins exhibited highly conserved motif compositions across all examined insect orders, with most sequences containing 15 identified motifs (from motif 1–15), except for Ephemeroptera, which lacked motif 13 and Lepidoptera lacking motif 9. Comparative analysis with vertebrate fibrillin-1, fibrillin-2, and fibrillin-3 showed that insect fibrillin-2 annotated proteins share high motif similarity with its vertebrate counterparts, indicative of a common ancestral origin (Fig. 2B). In contrast, annotated insect fibrillin-1 and fibrillin-3 proteins showed relatively low inter-order conservation, with extensive motif deletions or positional shifts. Their overall motif compositions differ significantly from both canonical vertebrate fibrillins and insect fibrillin-2 annotated proteins. For instance, fibrillin-1 annotated sequences from Diptera lack motifs 7, 11, 12, and 15, while those from Lepidoptera lack motif 7. For annotated fibrillin-3 proteins, only the C-terminal motifs 2, 4, 6 and 8 are relative conserved across species. In summary, annotated insect fibrillin members exhibit considerable variations in the number and arrangement of conserved motifs, which may underlie functional divergence among these protein groups.

### Typical structure domain analysis

**Figure 3:**
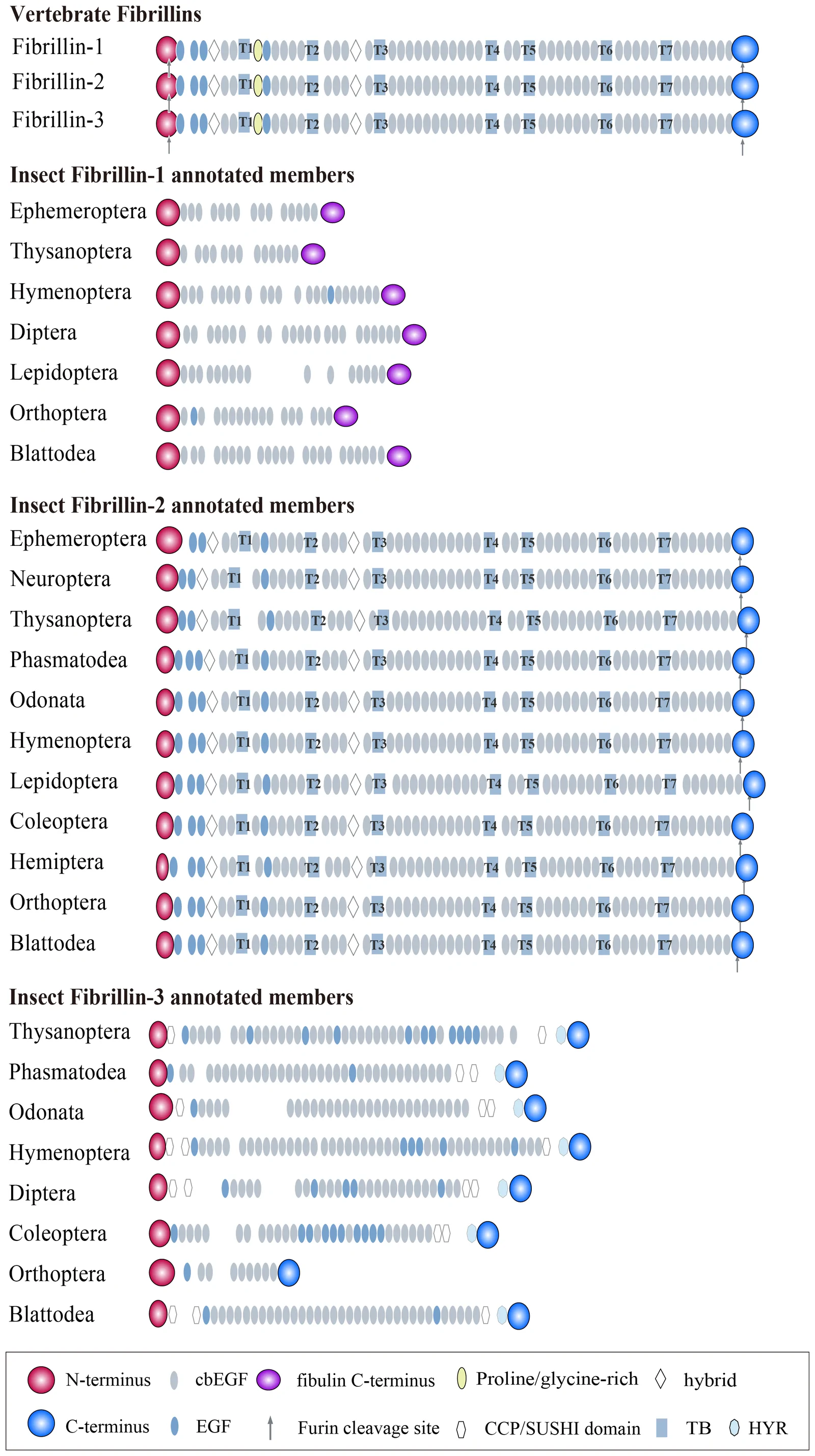
Overview of the domain structure of the vertebrate and insect fibrillin annotated proteins. Numbers within TB domains indicate the relative number of the respective domain within the fibrillin-2 of vertebrate and insect fibrillin-2 annotated members. Proprotein processing sites in the unique C-terminal domains (arrows) are indicated. Box shows the symbols used.

Structural domain analysis facilitates the identification of protein family affiliation and potential biological functions. Therefore, based on the functional domain profiles of fibrillin, we explored whether the three annotated insect fibrillin members possesses the canonical domain architecture of the fibrillin family. Structural domain analysis was conducted using InterPro (Fig. 3). The results demonstrated that insect annotated fibrillin-2 members exhibit a domain composition highly consistent with vertebrate fibrillins, consisting of EGF, TB and Hybrid domain. These members contain 48 EGF domains (including 44 cbEGF domains), seven TB domains and two Hybrid domains. The only structural difference from vertebrates fibrillin is that insect annotated fibrillin-2 proteins lack the specific proline/glycine-rich domain downstream of the first TB domain, which is substituted by a cbEGF-like domain. Additionally, annotated fibrillin-2 proteins from Ephemeroptera, Thysanoptera, and Neuroptera lack the N-terminal EGF domain found in other insect fibrillin-2 homologs, thereby possessing only 47 EGF domains. This variation may result from incomplete sequence assembly or deletion. Overall, annotated insect fibrillin-2 members share nearly identical structural characteristics with vertebrate fibrillins.

In contrast to annotated fibrillin-2 members, the insect sequences currently annotated as fibrillin-1 and fibrillin-3 in NCBI database lack the TB and hybrid domains that define canonical vertebrate fibrillins. Specifically, annotated fibrillin-1 members contain only EGF and cbEGF domains, along with a unique C-terminal fibulin-C domain, with domain numbers varying across species. Annotated fibrillin-3 proteins also lack typical TB and hybrid domains, and instead possess non-canonical fibrillins domains at the N- and C-termini, including Sushi/CCP and HYR domains.

### The specific R-X-K/R-R furin cleavage site is presented in the C-terminus of insect fibrillin-2 annotated members

**Figure 4:**
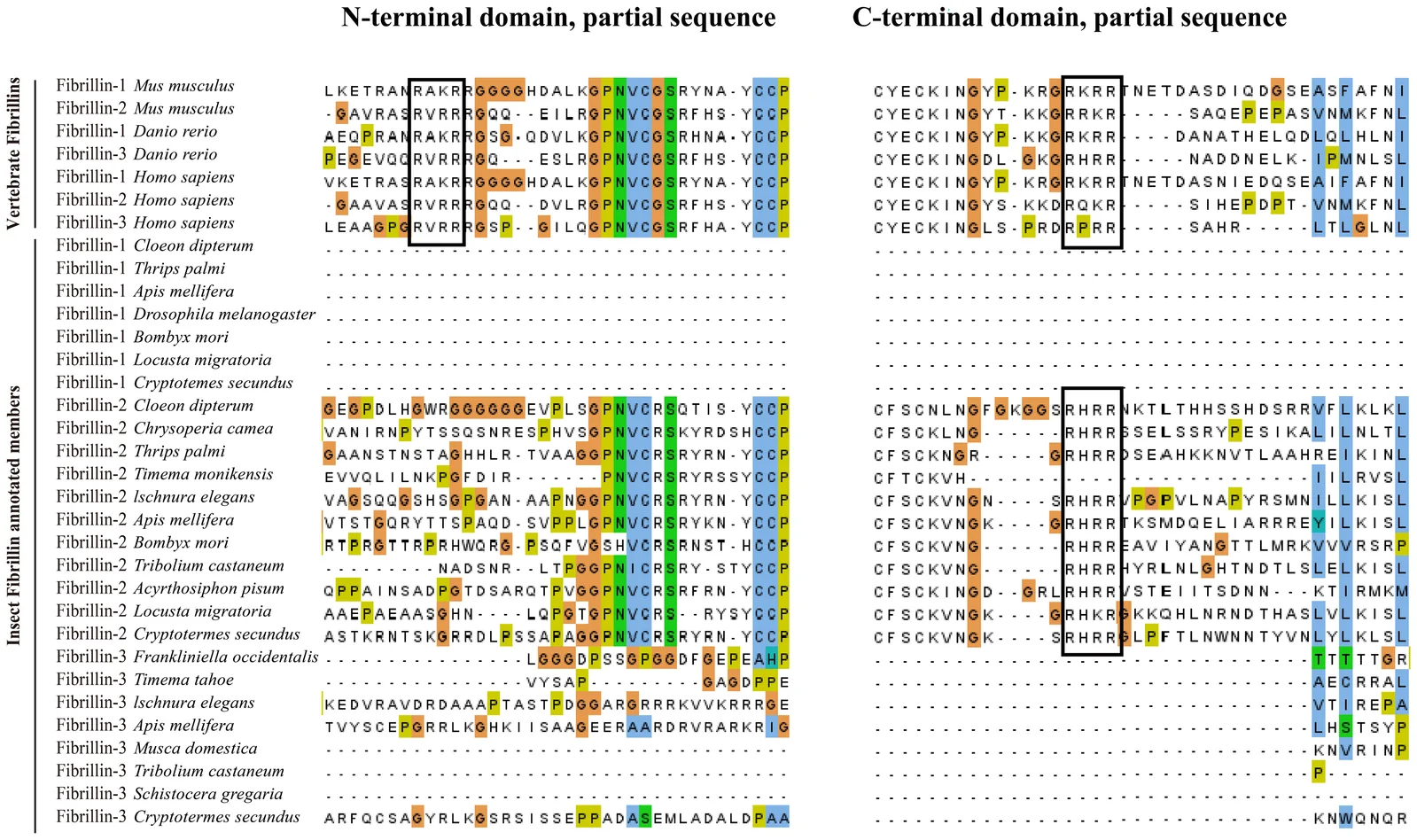
The N- and C-terminal of fibrillin proteins containing furin cleavage sites in vertebrates and some fibrillin-2 annotated members from insects. Boxed area represents furin cleavage site (R-X-K/R-R).

To further validate the familial affiliation of fibrillin sequences from 12 insect orders, furin cleavage sites were predicted using the online ProP 1.0 tool. The results revealed that among annotated insect fibrillin-1 members, furin cleavage site was predicted only in Thysanoptera, Orthoptera, and Blattodea species, and multiple sequence comparisons confirmed that these sites do not correspond to the conserved furin cleavage site in vertebrate fibrillins. For annotated fibrillin-2 members, although the flanking region of the vertebrate N-terminal cleavage site is relatively conserved in insect fibrillin-2 sequences, the canonical N-terminal cleavage site is absent. Instead, a highly conserved furin cleavage site (R-X-K/R-R) is exclusively present at its C-terminus. By contrast, no furin cleavage site were predicted in any annotated fibrillin-3 members (Fig. 4).

### Insect fibrillin-2 annotated members displayed similar spatial structures of the fibrillin family

**Figure 5:**
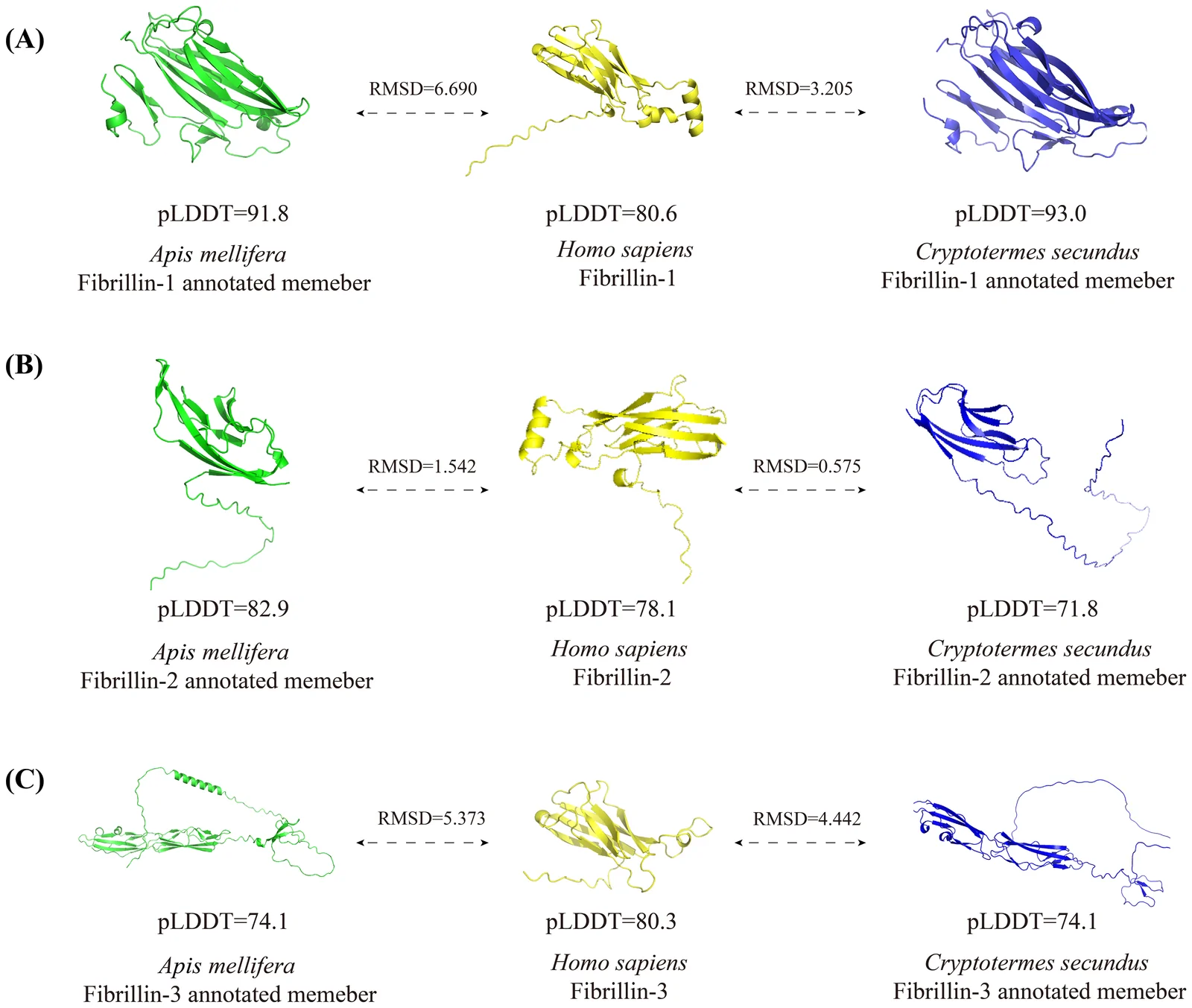
Similarity analysis of fibrillin-1 annotated members in insects and fibulin in vertebrates. (A) The phylogenetic tree was constructed using partial insect fibrillin-1 annotated proteins and vertebrate fibulin and fibrillin sequences. (B) C-terminal sequence alignment of insect fibrillin-1 annotated proteins and vertebrate fibulin 1–5. (C) Alignment of the predicted three-dimensional structure of the C terminus of *Locusta migratoria* fibrillin-1 annotated protein (slate colour) with that the *Homo sapiens* fibulin 1–5. Vert, vertebrate.

Given the high cross-species conservations of fibrillin C-terminal sequences, structural analysis of the C-terminal three-dimensional (3D) structure of insect fibrillin can facilitate sequence identification and classification. The AlphaFold2 tool was used to predict and analyze the C-terminal 3D structures of annotated fibrillin members from representative vertebrates and insect species. The structural analysis showed that vertebrate fibrillin-1 contains two *α*-helices, four *β*-turns and seven *β*-sheets; vertebrate fibrillin-2 contains one *α*-helix, five *β*-turns and seven *β*-sheets; and vertebrate fibrillin-3 contains seven *β*-turns and seven *β*-sheets, indicating that the seven *β*-sheets are highly conserved across vertebrate fibrillin families (Fig. S1). In insect, annotated fibrillin-1 members comprise one *α*-helix, ten *β*-turns, and eleven *β*-sheets. All annotated fibrillin-2 members retain seven *β*-sheets, while the number of *α*-helices and *β*-turns varies among species, ranging from zero to two for *α*-helices and three to five for *β*-turns. Annotated fibrillin-3 members possess eight *β*-turns and nineteen *β*-sheets, with two to three variable *α*-helices among different species (Figs. S2–S4). Collectively, insect fibrillin-2 annotated members exhibits the highest structural similarity to vertebrate fibrillins, particularly in the conserved seven *β*-sheets structure. In contrast, annotated insect fibrillin-1 and fibrillin-3 members present distinct predicted conformational characteristics. Furthermore, structural superimposition of insect and vertebrate fibrillin C-terminal domains revealed that only fibrillin-2 annotated members shows a well-matched spatial structure with the C-terminus region of human fibrillin (Fig. 5), whereas insect fibrillin-1 and fibrillin-3 annotated members exhibit substantially divergent structural conformations compared with human fibrillins.

### Identification of fibrillin-1 and fibrillin-3 annotated members in insects

**Figure 6:**
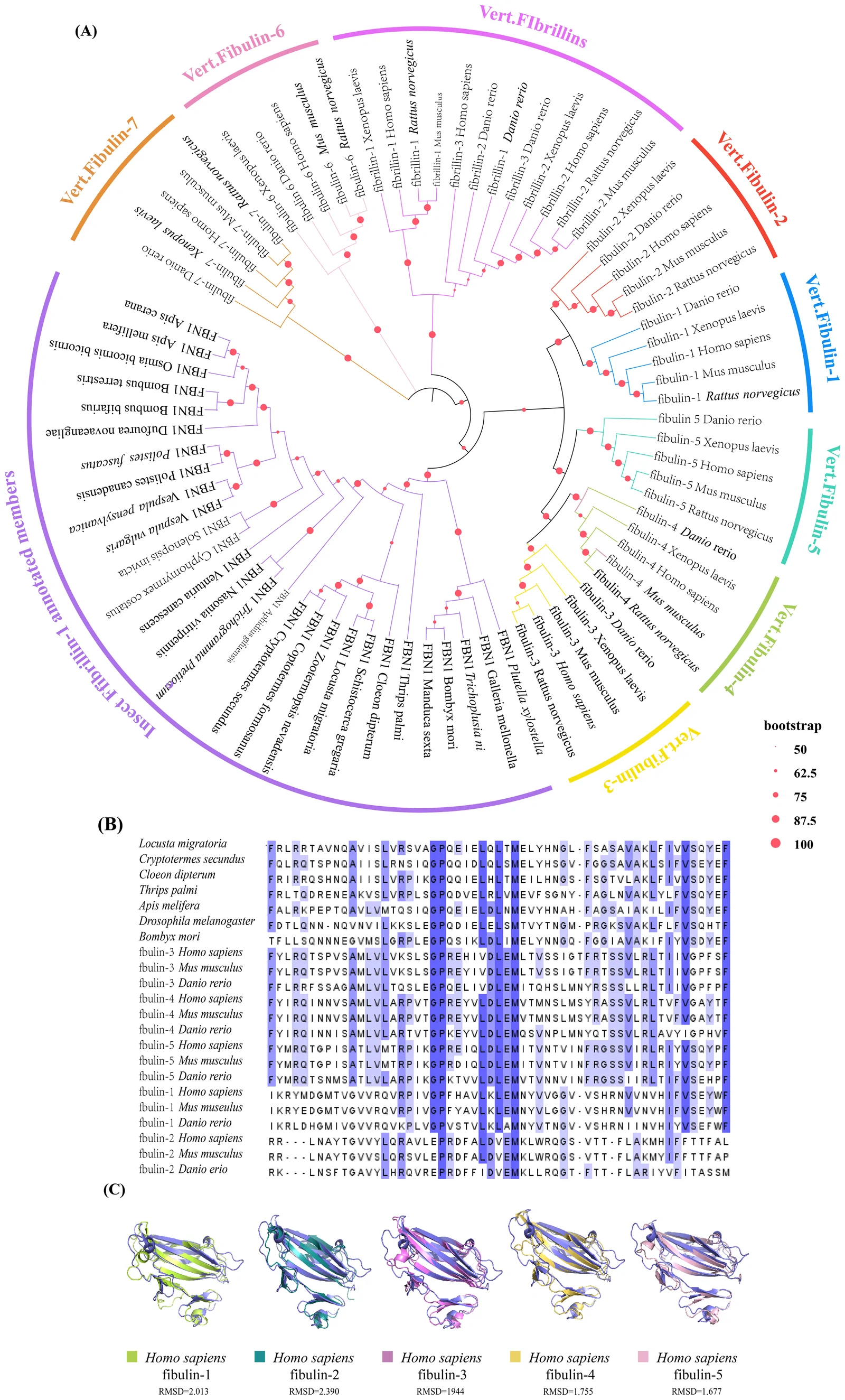
Prediction and superimposition of C-terminal structures between insect annotated fibrillins and their human homologs. (A) Prediction and superimposition of C-terminal structures between annotated fibrillin-1 from two insect species and their human homologs. (B) Prediction and superimposition of C-terminal structures between annotated fibrillin-2 from two insect species and their human homologs. (C) Prediction and superimposition of C-terminal structures between annotated fibrillin-3 from two insect species and their human homologs. The numbers represent the root mean square deviation of these structural superposition of insect and vertebrates. The left column (green colour) is the fibrillin annotated members of *Apis mellifera*, the middle (yellow colour) is the *Homo sapiens* fibrillin, and the right column (blue colour) is the fibrillin annotated members of *Cryptotermes secundus*. The protein backbones are depicted as ribbons showing the different secondary structure elements. The RMSD is a measure of the degree of distinction between two structures, with higher RMSD values indicating a larger degree of difference between the two structures.

**Figure 7:**
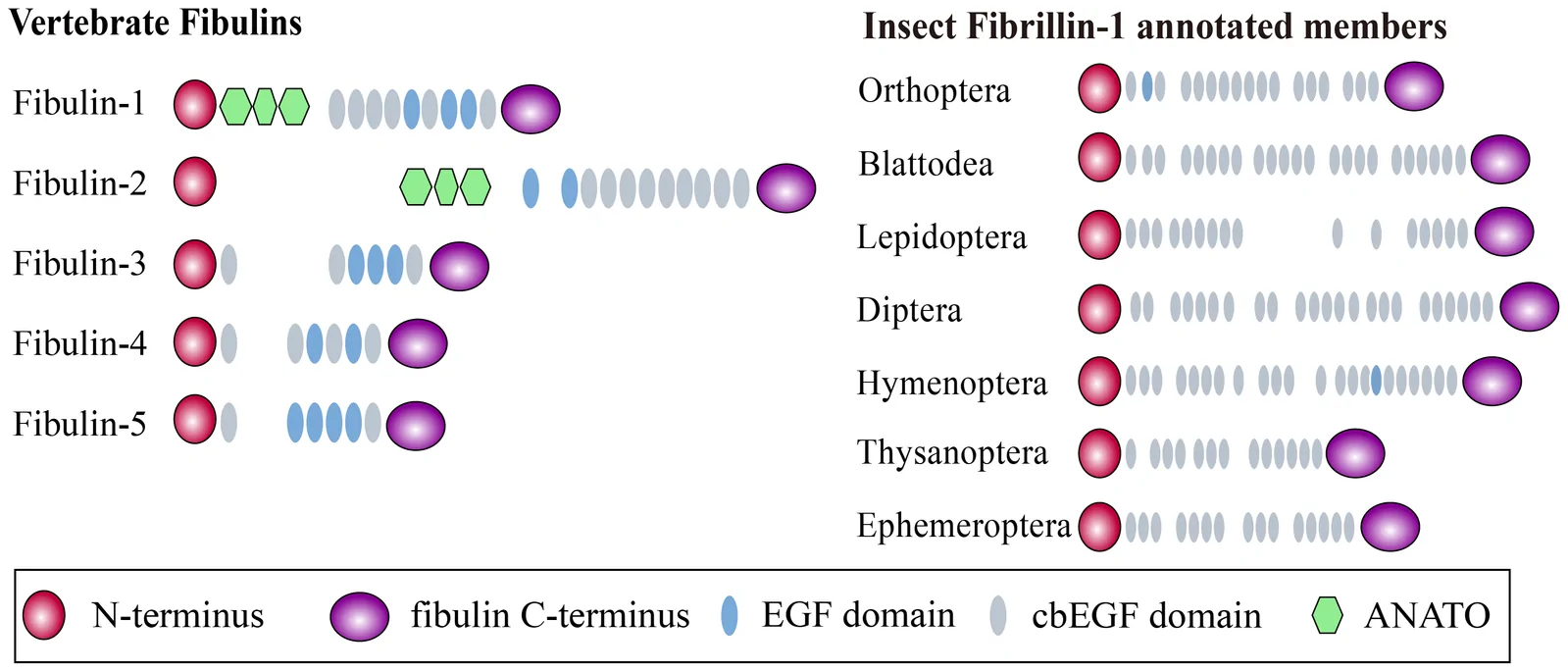
Overview of the domain structure of the vertebrate fibulin 1–5 and insect fibrillin-1 annotated members. Box shows the symbols used. ANATO, Anaphylotoxin-like domain.

In order to further clarify the evolutionary classification of NCBI-annotated insect fibrillin-1 and fibrillin-3, Smart BLAST was performed using annotated insect fibrillin-1 sequences as queries. The results revealed that the closely matched structural homologs correspond to vertebrate fibulins. Conventional NCBI protein BLAST demonstrated that insect fibrillin-1 annotated sequences exhibit the highest similarity to other insect fibrillin-1 homologs, while their best matches among vertebrate protein belong to the fibulin family. Consistently, vertebrate fibulin sequences yielded annotated insect fibrillin-1 annotated proteins as their top hits when searched against the insect species in the NCBI database. These findings indicate that annotated insect fibrillin-1 members share greater sequence and structural similarity with vertebrate fibulins than with canonical fibrillins. To further explore the evolutionary relationship between insect annotated fibrillin-1 members and fibulin, phylogenetic analysis was performed using 28 annotated insect fibrillin-1 sequences, together with fibulins and fibrillin sequences from *Homo sapiens*, *Rattus norvegicus*, *Mus musculus*, *Danio rerio*, and *Xenopus laevis.* The phylogenetic tree showed that annotated insect annotated fibrillin-1 sequences cluster tightly with vertebrate fibulin 1–5 rather than the fibrillin family, indicating a higher degree of homology (Fig. 6A). BLAST searches of human fibulin sequences against insect genome databases further identified annotated insect fibrillin-1 sequences as the best matches, supporting their close evolutionary relatedness. Furthermore, sequence alignment of the C-terminal regions of insect fibrillin-1 annotated members and vertebrate fibulin proteins revealed high cross-group conservation, further confirming their family similarity (Fig. 6B). In terms of domain architecture, annotated insect fibrillin-1 members possess the same EGF and fibulin_C domains as vertebrate fibulin 1–5 (Figs. 3 and 7). Collectively, these results demonstrate that NCBI-annotated insect fibrillin-1 proteins are evolutionarily closer to vertebrate fibulins than to fibrillins, with high sequence and structural identity to the fibulin family.

To further characterize this protein group, the C-terminal structures of annotated insect fibrillin-1 members and vertebrate fibulins were predicted. Structural comparison showed that both groups contain eleven *β*-sheets, multiple *α*-helices, and seven *β*-turns. Three-dimensional structural alignment of the optimal model of *Locusta migratoria* annotated fibulin-1 (pLDDT = 92.7) with vertebrate fibulin revealed that these insect fibrillin-1 annotated members overlaps well with vertebrate fibulin 1–5 (Fig. 6C), supporting the hypothesis that insect fibrillin-1 annotated members actually belong to the fibulin family.

In contrast, Smart BLAST searches for annotated insect fibrillin-3 members identified no homologous sequences or structural counterparts in vertebrates. Annotated fibrillin-3 proteins are distributed across eight insect orders (Thysanoptera, Phasmatodea, Odonata, Hymenoptera, Diptera, Coleoptera, Orthoptera, and Blattodea) and are detectable within these insect lineages, yet no close homologs exist in vertebrates. Structural domain analysis identified distinctive HYR and CCP domains in insect fibrillin-3. Subsequent homolog searches based on these characteristic domains failed to retrieve any previously proteins with identical domain architectures. These findings suggest that annotated insect fibrillin-3 members may represent an uncharacterized, insect-specific protein family.

## Discussion

The evolutionary relationships of vertebrate fibrillins have been well characterized (Piha-Gossack, Sossin & Reinhardt, 2012), whereas the annotation and evolution of invertebrate fibrillins remain poorly resolved. Previous studies have confirmed that insects encode only one genuine fibrillin protein (Piha-Gossack, Sossin & Reinhardt, 2012). However, the present study identified three distinct protein groups currently annotated as fibrillin in insects. Clarifying the classification and evolutionary relationships of insect fibrillin family members can resolve this inconsistency. Therefore, published vertebrate fibrillin sequences were used as queries to screen all insect sequences annotated as fibrillin. After removing redundant entries, phylogenetically distant outliers, and clearly erroneous sequences, a total of 373 putative insect fibrillin sequences were retained *via* sequence alignment and rigorous screening. The retrieved sequences covered 12 orders, including Ephemeroptera, Neuroptera, Thysanoptera, Phasmatodea, Odonata, Hymenoptera, Diptera, Lepidoptera, Coleoptera, Hemiptera, Orthoptera, and Blattodea. No fibrillin-related sequences were recovered from other insect orders, possibly due to the unavailability of published genomic data for those species. Multiple bioinformatics analyses were subsequently conducted on the collected sequences.

Human harbor three fibrillin family members. Fibrillin-1 is predominantly synthesized by mesenchymal cells and exhibits persistent expressed throughout lifespan (Summers et al., 2010; Davis et al., 2016), while fibrillin-2 and fibrillin-3 are mainly expressed during development stages (Davis & Summers, 2012). Fibrillin-1 synthesis is associated with late morphogenesis and the formation of mature organ structures, whereas fibrillin-2 synthesis coincides with early morphogenesis and the initiation of elastogenesis. Fibrillin-1 and fibrillin-2 exhibit partially overlapping functions and collectively mediate elastic tissue formation and homeostasis (Zhang, Hu & Ramirez, 1995; Ramirez & Sakai, 2010). By contrast, fibrillin-3 plays unique roles in bone growth and development (Sabatier et al., 2011). The present study revealed that insects encode only one genuine fibrillin homolog, corresponding to the currently annotated insect fibrillin-2. This finding likely reflects an ancestral evolutionary state of the fibrillin family in invertebrates. Insect fibrillin may serve as an ancestral gene that integrates the biological functions of vertebrates fibrillin-1 and fibrillin-2. Following the evolutionary emergence of elastin and the cartilage tissues in vertebrates, the ancestral fibrillin gene gradually diverged into three paralogs (fibrillin-1, fibrillin-2, and fibrillin-3), which subsequently evolved distinct functional characteristics.

In vertebrates, the cleavage of the unique N- and C- terminal sites of fibrillins regulates fibrillin-1 microfibril assembly (Milewicz et al., 1992). The processed fibrillin non only serves as a template for elastic fiber formation in elastic tissues, but also accumulates abundantly in non-elastin-expressing tissues (Kielty et al., 2002a; Kielty et al., 2002b). The C-terminal cleavage product of fibrillin, Asprosin, is primarily responsible for glucose regulation and appetite regulation (Romere et al., 2016; Godwin et al., 2019). In insects, however, the furin-cleavage site R-X-K/R-R is exclusively present at the C terminus of fibrillin-2, with no N-terminal cleavage site. This structural difference may drive functional and metabolic divergence of fibrillin between vertebrates and insects. Given that elastin is absent in invertebrates (Sage & Gray, 1979; Keeley Fred, 2013), it is speculated that processed insect fibrillin primarily participates in maintaining the elasticity and extensibility of the insect extracellular matrix. Structural comparison of fibrillin C-terminal regions between insects and humans revealed high similarities in both secondary and three-dimensional architectures. We hypothesize that furin-cleaved C-terminal products of insect fibrillin also participate in blood glucose and appetite regulation, although the underlying molecular mechanism remains to be further elucidated.

Insect fibrillin-1 may exert functions similar to those of vertebrate fibulins. Multiple lines of evidence suggest that insect fibrillin-1 is structurally and sequentially more homologous to fibulin than to canonical fibrillins. The fibulin family consists of secreted glycoproteins that associate with basement membranes, elastic fibers, and other matrices. This family includes seven members, all characterized by conserved EGF-like domain (nine in fibulin-1, eleven in fibulin-2, and six in fibulin-3, -4, and -5) and a unique C-terminal fibulin domain (Timpl et al., 2003; De Vega, Iwamoto & Yamada, 2009). Fibulin-1 and fibulin-2 display distinct yet overlapping expression and molecular interaction patterns. Both localize to basement membranes, elastic fibers, and other connective tissue and participate in the assembly of diverse extracellular supramolecular structures (Reinhardt et al., 1996; Zhang et al., 1996; Segade, 2010). Fibulin-3, fibulin-4 and fibulin-5 are chordate-specific proteins and play crucial roles in elastic fiber formation, extracellular matrix interactions, and tissue development (Argraves et al., 1989; Yanagisawa et al., 2002; Wagenseil & Mecham, 2007). Consistently, all collected insect sequences annotated as fibrillin-1 possess typical EGF domain and a C-terminal fibulin domain. However, the number of EGF domains varies considerably across insect among orders, ranging from only 13 in Thysanoptera to a maximum of 23 in Blattodea. In contrast, the EGF domain count of mammalian fibulins is relatively conserved. Such interspecific variations may result from distinct living environments and physiological constraints, driving domain rearrangement and modification in insect fibulin homologs to accommodate lineage-specific functional requirements during evolution.

In *Caenorhabditis elegans*, fibulin bind to basement membranes and regulate cell adhesion, supporting a metazoan origin of this family (Kubota, Kuroki & Nishiwaki, 2004; Segade, 2010). As intermediate evolutionary lineages between nematodes and vertebrates, insects are hypothesized to retain the fundamental functions of the fibulin family. Specifically, insect fibulin homologs may participate in extracellular matrix assembly and stabilization, and engage in multiple biological processes, including adhesion and tissue organ development.

Fibrillin-3 is not a bona fide member of the fibrillin family and possesses unique HYR and CCP domains. The HYR domain is an extracellular domain consisting of approximately 80–100 amino acids that adopts an immunoglobulin-like fold. Found in multiple eukaryotic proteins, this domain typically appears alongside complement control protein modules or in tandem repeats and participates in cell surface adhesion (Wessel et al., 1998; Callebaut et al., 2000). The CCP domain is defined by a conserved consensus sequence of around 60 amino acids (Reid & Day, 1989; Ojha et al., 2019). In CCP-containing proteins, this domain mediates protein-protein interaction and contributes to complement system regulation and blood coagulation (De Vega et al., 2007). It is widely distributed not only in complement-activating regulatory factors (Chou & Heinrikson, 1997; Morgan et al., 2012; Ojha et al., 2019), but also in diverse proteins involved in complement activation (Morikis & Lambris, 2005), cell adhesion (Williams & Barclay, 1988), and blood clotting. To date, no previously characterized protein exhibits a domain architecture identical to that of annotated insect fibrillin-3. Given the functional properties of its unique domain, fibrillin-3 annotated proteins are speculated to participate in cell surface adhesion, though its precise biological functions remain to be further investigated.

In summary, only insect protein annotated as fibrillin-2 are bona fide members of the fibrillin family, while insect sequences currently annotated as fibrillin-1 and fibrillin-3 are likely misannotated. Insect annotated fibrillin-1 is clearly defined herein as fibulin homolog, given its domain architecture consisting of EGF and fibulin-C domains. Unlike vertebrates that possess multiple fibrillin paralogs, insects harbor only one genuine fibrillin protein, whereas their annotated fibrillin-1 homologs are phylogenetically closer to vertebrate fibulins. Accordingly, protein featuring canonical fibrillin domain combinations (EGF, TB and Hybrid domains) should be correctly annotated as fibrillins. In contrast, annotated insect fibrillin-3 sequences, which contain unique HYR and CCP domains are tentatively reclassified as putative cell-adhesion-related-proteins. Nevertheless, whether insect fibrillin-2 participates in elastic tissue assembly and glucose metabolism remains to be comprehensively investigated, and the precise biological functions of insect fibrillin-1 and fibrillin-3 require further exploration.

## Conclusion

In conclusion, the present study systematically collected and comprehensively analyzed 373 fibrillin-annotated protein sequences from 12 insect orders, resolving the long-standing annotation ambiguity surrounding insect fibrillin-related proteins *via* combined phylogenetic analysis, sequence characterization, conserved motif and domain profiling, as well as three-dimensional structural comparison. The results verify that among the three insect entries annotated as fibrillin-1, fibrillin-2, and fibrillin-3 in public databases, only proteins annotated as fibrillin-2 are orthologs of the canonical vertebrate fibrillin family; these sequences maintain a conserved cysteine proportion, canonical domain architecture (EGF, TB, and Hybrid domains), a conserved C-terminal furin cleavage motif, and a C-terminal tertiary structure with high structural superimposition matching vertebrate fibrillins. By contrast, insect proteins annotated as fibrillin-1 cluster phylogenetically and structurally with the vertebrate fibulin family instead of fibrillins, whereas those annotated as insect fibrillin-3 likely constitute an uncharacterized insect-specific protein family implicated in cell adhesion. These two protein groups share close evolutionary relatedness to the fibrillin family and have therefore been subjected to erroneous annotation across public databases.

##  Supplemental Information

10.7717/peerj.21577/supp-1 Supplemental Information 1 Supplemental Figures and Tables. All four supplementary figures (Figure S1–S4) and one supplementary table (Table S1), which lists all protein sequences analyzed in this study.

10.7717/peerj.21577/supp-2 Supplemental Information 2 Graphical abstract. The graphical abstract figure of this study together with its descriptive captions.

## Additional Information and Declarations

### Data Availability

The following information was supplied regarding data availability: The data is available in the Supplementary File.
