# Supplementary material for: Identification and evolutionary analysis of insect fibrillin with their closely related family proteins
Source: PeerJ. 2026 Aug 24;14:e21577. doi: 10.7717/peerj.21577 (PMC13525746; doi:10.7717/peerj.21577)
Supplement: Supplementary file 1 [file peerj-14-21577-s001.docx]

Supporting Information:

**Figure S1. Schematic diagram for sequence conservation and secondary structure analysis of vertebrate fibrillin-1 protein**. Fibrillin-1 from *Homo sapiens* was selected as the example for secondary structure presentation.

**Figure S2. Schematic diagram for sequence conservation and secondary structure analysis of insect** **fibrillin-1 annotated protein**. Fibrillin-1 annotated protein from *Locusta migratoria* was selected as the example for secondary structure presentation.

**Figure S3. Schematic diagram for sequence conservation and secondary structure analysis of insect fibrillin-2 annotated protein**. Fibrillin-2 annotated protein from *Locusta migratoria* was selected as the example for secondary structure presentation.

**Figure S4. Schematic diagram for sequence conservation and secondary structure analysis of insect fibrillin-3 annotated protein**. Fibrillin-3 annotated protein from *Tribolium castaneum* was selected as the example for secondary structure presentation.

Table S1. Sequences analyzed in this study.

| Order | Family | Genus | Species | Fibrillin-1 | Fibrillin-2 | Fibrillin-3 |
| --- | --- | --- | --- | --- | --- | --- |
| Ephemeroptera | Ephemeridae | Ephemera | *Ephemera danica* |  | KAF4525808.1 |  |
|  | Baetidae | Cloeon | *Cloeon dipterum* | CAB3381955.1 | CAB3384638.1 |  |
| Neuroptera | Chrysopidae | Chrysoperla | *Chrysoperla carnea* |  | XP_044739911.1 |  |
| Thysanoptera | Thripidae | Thrips | *Thrips palmi* | XP_034240918.1 | XP_034242198.1 | XP_034254429.1 |
|  |  | Frankliniella | *Frankliniella occidentalis* |  | XP_052120881.1 | XP_052119706.1 |
| Phasmatodea | Timematidae | Timema | *Timema shepardi* |  | CAD7256503.1 | CAD7268779.1 |
|  |  |  | *Timema genevievae* |  | CAD7591982.1 |  |
|  |  |  | *Timema poppensis* |  | CAD7397684.1 |  |
|  |  |  | *Timema monikensis* |  | CAD7423795.1 | CAD7433908.1 |
|  |  |  | *Timema tahoe* |  | CAD7455269.1 | CAD7456482.1 |
|  |  |  | *Timema douglasi* |  | CAD7197318.1 |  |
|  |  |  | *Timema cristinae* |  | CAD7393933.1 | CAD7398207.1 |
|  |  |  | *Timema californicum* |  | CAD7569780.1 |  |
|  |  |  | *Timema bartmani* |  | CAD7439563.1 |  |
| Odonata | Coenagrionidae | Ischnura | *Ischnura elegans* |  | XP_046399830.1 | XP_046390338.1 |
|  | Libellulidae | Ladona | *Ladona fulva* |  | KAG8223124.1 |  |
| Hymenoptera | Cephidae | Cephus | *Cephus cinctus* | XP_015599681.1 | XP_015608281.1 | XP_015605338.1 |
|  | Megachilidae | Osmia | *Osmia bicornis* | XP_029040591.2 | XP_046143978.1 | XP_029052478.2 |
|  |  |  | *Osmia lignaria* | XP_034188272.1 | XP_034176025.1 | XP_034176710.1 |
|  |  | Megachile | *Megachile rotundata* | XP_012149838.1 | XP_003707730.2 | XP_012149877.1 |
|  | Halictidae | Nomia | *Nomia melanderi* | XP_031834529.1 | XP_031849971.1 | XP_031838240.1 |
|  |  | Dufourea | *Dufourea novaeangliae* | XP_015437838.1 | XP_015435488.1 | XP_015438741.1 |
|  | Tenthredinidae | Athalia | *Athalia rosae* | XP_048511323.1 | XP_025602128.2 | XP_048506852.1 |
|  | Formicidae | Nylanderia | *Nylanderia fulva* | XP_029167576.1 | XP_029164594.1 | XP_029163192.1 |
|  |  | Harpegnathos | *Harpegnathos saltator* | XP_011145491.1 | XP_011149570.1 | XP_025154714.1 |
|  |  | Dinoponera | *Dinoponera quadriceps* | XP_014488735.1 | XP_014481820.1 | XP_014484374.1 |
|  |  | Odontomachus | *Odontomachus brunneus* | XP_032679006.1 | XP_032678016.1 | XP_032691386.1 |
|  |  | Monomorium | *Monomorium pharaonis* | XP_012529953.2 | XP_036142453.1 |  |
|  |  | Temnothorax | *Temnothorax longispinosus* |  | TGZ47644.1 |  |
|  |  |  | *Temnothorax curvispinosus* | XP_024879851.1 | XP_024871130.1 | XP_024880984.1 |
|  |  | Camponotus | *Camponotus floridanus* | XP_025262768.1 | XP_011267661.2 | XP_025267672.1 |
|  |  | Acromyrmex | *Acromyrmex charruanus* | KAG5348281.1 | KAG5335939.1 | KAG5343895.1 |
|  |  |  | *Acromyrmex insinuator* | KAG5311759.1 | KAG5315480.1 | KAG5307441.1 |
|  |  |  | *Acromyrmex echinatior* | XP_011056974.1 | XP_011064414.1 | XP_011057360.1 |
|  |  |  | *Acromyrmex heyeri* | KAG5317087.1 | KAG5326852.1 | KAG5330507.1 |
|  |  | Cataglyphis | *Cataglyphis hispanica* | XP_050458174.1 | XP_050461222.1 | XP_050459409.1 |
|  |  | Formica | *Formica exsecta* | XP_029659137.1 | XP_029674885.1 | XP_029666283.1 |
|  |  | Solenopsis | *Solenopsis invicta* | XP_025987458.1 | XP_025993785.1 | XP_039302308.1 |
|  |  | Atta | *Atta colombica* | XP_018054614.1 | XP_018045586.1 | XP_018044449.1 |
|  |  |  | *Atta cephalotes* | XP_012059964.1 | XP_012058472.1 | XP_012062443.1 |
|  | Apidae | Habropoda | *Habropoda laboriosa* | XP_017792648.1 | XP_017795563.1 | XP_017797388.1 |
|  |  | Eufriesea | *Eufriesea mexicana* | XP_017758004.1 | XP_017754869.1 | XP_017760224.1 |
|  |  | Melipona | *Melipona quadrifasciata* | KOX81084.1 | KOX76465.1 |  |
|  |  | Ceratina | *Ceratina calcarata* | XP_017892365.1 | XP_026667499.1 | XP_017876994.1 |
|  |  | Bombus | *Bombus pyrosoma* | XP_043604994.1 | XP_043593182.1 | XP_043584964.1 |
|  |  |  | *Bombus vosnesenskii* | XP_033361800.1 | XP_033348717.1 | XP_033364106.1 |
|  |  |  | *Bombus impatiens* | XP_024228038.1 | XP_003491105.2 | XP_024224615.2 |
|  |  |  | *Bombus bifarius* | XP_033310494.1 | XP_033314049.1 | XP_033318623.1 |
|  |  |  | *Bombus pascuorum* | XP_060818231.1 | XP_060815651.1 | XP_060813908.1 |
|  |  |  | *Bombus terrestris* | XP_003402231.2 | XP_003399227.3 | XP_020723285.2 |
|  |  | Apis | *Apis florea* | XP_012346769.1 | XP_003692245.2 | XP_031771390.1 |
|  |  |  | *Apis laboriosa* | XP_043800779.1 | XP_043798235.1 | XP_043784301.1 |
|  |  |  | *Apis dorsata* | XP_006623621.1 | XP_006615130.1 | XP_031364843.1 |
|  |  |  | *Apis cerana* | XP_016915008.1 | XP_016908651.1 | XP_016912962.1 |
|  |  |  | *Apis mellifera* | XP_006558164.2 | XP_003250904.3 | XP_006561358.2 |
|  | Vespidae | Polistes | *Polistes dominula* | XP_015191223.1 | XP_015191403.1 | XP_015181156.1 |
|  |  |  | *Polistes canadensis* | XP_014611555.1 | XP_015191403.1 | XP_014598460.1 |
|  |  |  | *Polistes fuscatus* | XP_043493548.1 | XP_043495263.1 | XP_043503452.1 |
|  |  | Vespula | *Vespula pensylvanica* | XP_043673952.1 | XP_043673249.1 | XP_043666048.1 |
|  |  |  | *Vespula vulgaris* | XP_050857723.1 | XP_050857417.1 | XP_050846925.1 |
|  |  | Vespa | *Vespa mandarinia* | XP_035743414.1 | XP_035743589.1 | XP_035730081.1 |
|  | Ichneumonidae | Venturia | *Venturia canescens* | XP_043279235.1 | XP_043284259.1 | XP_043277768.1 |
| Diptera | Stratiomyidae | Hermetia | *Hermetia illucens* | XP_037925719.1 |  | CAD7082562.1 |
|  | Diopsidae | Teleopsis | *Teleopsis dalmanni* | XP_037942354.1 |  | XP_037938996.1 |
|  | Cecidomyiidae | Contarinia | *Contarinia nasturtii* | XP_031626997.1 |  | XP_031619499.1 |
|  |  | Sitodiplosis mosellana | *Sitodiplosis mosellana* | XP_055297571.1 |  | XP_055324777.1 |
|  | Sciaridae | Bradysia | *Bradysia odoriphaga* |  |  | KAG4071630.1 |
|  |  |  | *Bradysia coprophila* | XP_037034078.1 |  | XP_037034818.1 |
|  | Sarcophagidae | Sarcophaga | *Sarcophaga bullata* |  |  | TMW51781.1 |
|  | Calliphoridae | Lucilia | *Lucilia cuprina* | XP_046802705.1 |  | XP_046807654.1 |
|  | Muscidae | Musca | *Musca domestica* |  |  | XP_011292947.1 |
|  | Drosophilidae | Drosophila | *Drosophila melanogaster* | NP_001284712.1 |  | NP_001137905.1 |
|  |  |  | *Drosophila santomea* | XP_039496577.1 |  | XP_039487701.1 |
|  |  |  | *Drosophila teissieri* | XP_043657521.1 |  | XP_043647691.1 |
|  |  |  | *Drosophila simulans* | XP_039152063.1 |  | XP_016030815.1 |
|  |  |  | *Drosophila sechellia* | XP_032579828.1 |  | XP_032574632.1 |
|  |  |  | *Drosophila erecta* | XP_026839234.1 |  | XP_026833391.1 |
|  |  |  | *Drosophila mauritiana* | XP_033168224.1 |  | XP_033160083.1 |
|  | Tephritidae | Rhagoletis | *Rhagoletis zephyria* | XP_017467398.1 |  | XP_017470399.1 |
|  |  |  | *Rhagoletis pomonella* | XP_036334772.1 |  | XP_036338309.1 |
|  |  | Zeugodacus | *Zeugodacus cucurbitae* | XP_011193667.1 |  | XP_028901941.2 |
|  |  |  | *Bactrocera oleae* | XP_036222945.1 |  | XP_014093449.1 |
|  |  | Ceratitis | *Ceratitis capitata* | XP_020712926.2 |  | XP_012157231.1 |
|  | Psychodidae | Phlebotomus | *Phlebotomus papatasi* | XP_055704020.1 |  | XP_055703294.1 |
|  |  | Lutzomyia | *Lutzomyia longipalpis* | XP_055690308.1 |  | XP_055688516.1 |
|  | Culicidae | Culex | *Culex quinquefasciatus* | XP_038110721.1 |  | XP_038117532.1 |
|  |  | Anopheles | *Anopheles nili* | XP_053671043.1 |  | XP_053674471.1 |
|  |  | Aedes | *Aedes aegypti* | XP_021695535.1 |  | XP_021695535.1 |
|  | Chironomidae | Chironomus | *Chironomus riparius* | CAG9807748.1 |  | CAG9801622.1 |
| Lepidoptera | Cosmopterigidae | Hyposmocoma | *Hyposmocoma kahamanoa* | XP_026317307.1 |  |  |
|  | Gelechiidae | Tuta | *Tuta absoluta* | KAJ2941069.1 | KAJ2949364.1 |  |
|  | Lycaenidae | Aricia | *Aricia agestis* |  | XP_041980391.1 |  |
|  | Plutellidae | Plutella | *Plutella xylostella* | XP_048487046.1 | XP_048484593.1 |  |
|  | Papilionidae | Parnassius | *Parnassius apollo* |  | CAG4985242.1 |  |
|  |  |  | *Parnassius mnemosyne* | CAK1595570.1 | CAK1580765.1 |  |
|  |  | Papilio | *Papilio machaon* | XP_045539601.1 | XP_045538865.1 |  |
|  |  |  | *Papilio xuthus* | XP_013168797.1 | XP_013169621.1 |  |
|  | Crambidae | Chilo | *Chilo suppressalis* |  | CAH2981978.1 |  |
|  |  | Ostrinia | *Ostrinia furnacalis* |  | XP_048484593.1 |  |
|  | Erebidae | Arctia | *Arctia plantaginis* | CAB3255053.1 | CAB3260460.1 |  |
|  | Nymphalidae | Maniola | *Maniola hyperantus* | XP_034837021.1 | XP_034833316.1 |  |
|  |  |  | *Maniola jurtina* | XP_045780219.1 | XP_045776008.1 |  |
|  |  | Pararge | *Pararge aegeria aegeria* | CAH2243695.1 | XP_039758394.1 |  |
|  |  | Melitaea | *Melitaea cinxia* | XP_045456448.1 | XP_045450412.1 |  |
|  |  | Nymphalis | *Nymphalis io* | XP_050356973.1 | XP_050350105.1 |  |
|  |  | Vanessa | *Vanessa tameamea* | XP_026493342.1 |  |  |
|  |  |  | *Vanessa cardui* |  | XP_046966467.1 |  |
|  |  |  | *Vanessa atalanta* | XP_047540623.1 | XP_047532817.1 |  |
|  |  | Danaus | *Danaus chrysippus* | CAG9570362.1 | CAG9565842.1 |  |
|  | Pyralidae | Amyelois | *Amyelois transitella* | XP_013200140.1 | XP_013192035.1 |  |
|  |  | Galleria | *Galleria mellonella* | XP_052752971.1 | XP_052748019.1 |  |
|  |  | Plodia | *Plodia interpunctella* | XP_053618965.1 | XP_053611317.1 |  |
|  | Sphingidae | Manduca | *Manduca sexta* | XP_030021778.2 | XP_037292480.1 |  |
|  | Pieridae | Leptidea | *Leptidea sinapis* |  | XP_050679493.1 |  |
|  |  | Colias | *Colias croceus* | XP_045507392.1 | XP_045501476.1 |  |
|  |  | Zerene | *Zerene cesonia* | XP_038219182.1 | XP_038222121.1 |  |
|  |  | Pieris | *Pieris macdunnoughi* | CAF4901842.1 | CAF4921779.1 |  |
|  |  |  | *Pieris napi* | XP_047519048.1 | XP_047505847.1 |  |
|  |  |  | *Pieris rapae* | XP_022130951.2 | XP_022129966.2 |  |
|  |  |  | *Pieris brassicae* |  | XP_045512045.1 |  |
|  | Noctuidae | Helicoverpa | *Helicoverpa armigera* | XP_049705721.1 | XP_049702073.1 |  |
|  |  |  | *Helicoverpa zea* | XP_047037504.1 | XP_047028848.1 |  |
|  |  | Trichoplusia | *Trichoplusia ni* | XP_026730463.1 | XP_026740926.1 |  |
|  |  | Spodoptera | *Spodoptera litura* | XP_022835807.1 | XP_022824896.1 |  |
|  |  |  | *Spodoptera frugiperda* |  | XP_035447023.2 |  |
|  |  |  | *Spodoptera exigua* | CAH0697625.1 | CAH0687614.1 |  |
|  |  | Bombyx | *Bombyx mandarina* | XP_028044273.1 | XP_028026554.1 |  |
|  |  |  | *Bombyx mori* | XP_037876845.1 | XP_037874370.1 |  |
| Coleoptera | Elateridae | Ignelater | *Ignelater luminosus* |  | KAF2900037.1 | KAF2901586.1 |
|  | Buprestidae | Agrilus | *Agrilus planipennis* |  | XP_018328224.1 | XP_025835536.1 |
|  | Cerambycidae | Anoplophora | *Anoplophora glabripennis* |  | XP_018574078.1 | XP_018575830.1 |
|  | Nitidulidae | Aethina tumida | *Aethina tumida* |  | XP_049820430.1 | XP_019864843.2 |
|  | Silphidae | Nicrophorus | *Nicrophorus vespilloides* |  | XP 017775669.1 | XP 017775137.1 |
|  | Coccinellidae | Harmonia | *Harmonia axyridis* |  | XP_045465550.1 |  |
|  |  | Coccinella | *Coccinella septempunctata* |  | XP_044756027.1 |  |
|  | Chrysomelidae | Gonioctena | *Gonioctena quinquepunctata* |  | KAG5873658.1 | KAG5877748.1 |
|  |  | Diabrotica | *Diabrotica virgifera* |  | XP_028134634.1 | XP_050517703.1 |
|  | Tenebrionidae | Tribolium | *Tribolium madens* |  | XP_044258529.1 | XP_044269949.1 |
|  |  |  | *Tribolium castaneum* |  | XP_008201364.1 | XP_008199507.1 |
|  |  | Tenebrio | *Tenebrio molitor* |  | CAH1374115.1 | KAJ3629595.1 |
|  | Scarabaeidae | Oryctes | *Oryctes borbonicus* |  | KRT83824.1 |  |
|  |  | Onthophagus | *Onthophagus taurus* |  | XP_022906889.1 | XP_022910351.1 |
|  | Lampyridae | Abscondita | *Abscondita terminalis* |  | KAF5275832.1 | KAF5270999.1 |
|  |  | Lamprigera | *Lamprigera yunnana* |  | KAF5305440.1 | KAF5278971.1 |
|  |  | Photinus | *Photinus pyralis* |  | XP_031338300.1 | XP_031347962.1 |
|  | Curculionidae | Rhynchophorus | *Rhynchophorus ferrugineus* |  | KAF7284742.1 | KAF7283330. |
|  |  | Dendroctonus | *Dendroctonus ponderosae* |  | XP_019761701.2 | XP_048521028.1 |
|  |  | Sitophilus | *Sitophilus oryzae* |  | XP_030760954.1 | XP_030758366.1 |
| Hemiptera | Pentatomidae | Halyomorpha | *Halyomorpha halys* |  | XP_024214767.1 |  |
|  | Miridae | Nesidiocoris | *Nesidiocoris tenuis* |  | BES94751.1 |  |
|  |  | Apolygus | *Apolygus lucorum* |  | KAF6210291.1 |  |
|  | Cicadellidae | Homalodisca | *Homalodisca vitripennis* |  | XP_046677960.1 |  |
|  | Liviidae | Diaphorina | *Diaphorina citri* |  | KAI5696491.1 |  |
|  | Delphacidae | Laodelphax | *Laodelphax striatellus* |  | RZF38291.1 |  |
|  |  | Nilaparvata | *Nilaparvata lugens* |  | XP_039291554.1 |  |
|  | Cimicidae | Cimex | *Cimex lectularius* |  | XP_014244418.1 |  |
|  | Phylloxeridae | Daktulosphaira | *Daktulosphaira vitifoliae* |  | XP_050520391.1 |  |
|  | Aleyrodidae | Bemisia | *Bemisia tabaci* |  | XP_018912347.1 |  |
|  | Aphididae | Melanaphis sacchari | *Melanaphis sacchari* |  | XP_025200878.1 |  |
|  |  | Cinara | *Cinara cedri* |  | VVC40262.1 |  |
|  |  | Sipha | *Sipha flava* |  | XP_025412033.1 |  |
|  |  | Diuraphis | *Diuraphis noxia* |  | XP_015369559.1 |  |
|  |  | Aphis | *Aphis craccivora* |  | KAF0759613.1 |  |
|  |  |  | *Aphis glycines* |  | KAE9545670.1 |  |
|  |  |  | *Aphis gossypii* |  | XP_050062052.1 |  |
|  |  | Rhopalosiphum | *Rhopalosiphum maidis* |  | XP_026815477.1 |  |
|  |  |  | *Rhopalosiphum padi* |  | XP_060837258.1 |  |
|  |  | Myzus | *Myzus persicae* |  | XP_022176674.1 |  |
|  |  | Acyrthosiphon | *Acyrthosiphon pisum* |  | XP_001945971.2 |  |
| Orthoptera | Acrididae | Locusta | *Locusta migratoria* | LOCMI12474 | LOCMI08369 | LOCMI03845 |
|  |  | Schistocerca | *Schistocerca gregaria* | XP_049844465.1 | XP_049837718.1 | XP_049829990.1 |
|  |  |  | *Schistocerca piceifrons* | XP_047103942.1 | XP_047121364.1 | XP_047115483.1 |
|  |  |  | *Schistocerca americana* | XP_046985786.1 | XP_047004426.1 | XP_046980990.1 |
|  |  |  | *Schistocerca serialis cubense* | XP_049954284.1 | XP_049937531.1 | XP_049951288.1 |
|  |  |  | *Schistocerca nitens* | XP_049805270.1 | XP_049790862.1 | XP_049800974.1 |
|  |  |  | *Schistocerca cancellata* | XP_049782103.1 | XP_049764774.1 | XP_049774892.1 |
|  | Gryllidae | Gryllus | *Gryllus bimaculatus* | GLG97386.1 |  |  |
| Blattodea | Termopsidae | Zootermopsis | *Zootermopsis nevadensis* | XP_021928588.1 | XP_021926107.1 | XP_021934108.1 |
|  | Kalotermitidae | Cryptotermes | *Cryptotermes secundus* | XP_023706876.1 | XP_023706358.1 | XP_023722246.1 |
|  | Rhinotermitidae | Coptotermes | *Coptotermes formosanus* | GFG28443.1 | GFG40449.1 | GFG35884.1 |
|  | Blattidae | Periplaneta | *Periplaneta americana* |  | KAJ4433094.1 |  |
