# Supplementary material for: Identification and evolutionary analysis of insect fibrillin with their closely related family proteins
Source: PeerJ. 2026 Aug 24;14:e21577. doi: 10.7717/peerj.21577 (PMC13525746; doi:10.7717/peerj.21577)
Supplement: Supplementary file 2 [file peerj-14-21577-s002.docx]

**Graphical abstract**

Fibrillins comprise a class of extracellular matrix proteins, and their C-terminal peptides function in the regulation of glucose metabolism in vertebrates. In this study, Chen et al. collected all annotated fibrillin family protein sequences of insect species available in the NCBI database. Analyses of phylogeny, sequence features, conserved motifs and domains, as well as three-dimensional structures showed that only annotated insect fibrillin-2 homologs are genuine fibrillin family members with markedly different evolutionary patterns from those of vertebrate fibrillins. Proteins annotated as fibrillin-1 are more closely related to vertebrate fibulins, whereas those annotated as fibrillin-3 represent a previously uncharacterized protein family associated with cell adhesion. These two groups are closely related to fibrillin family proteins and have been misannotated in public databases.
